# Serum Metabolomic Response to Long-Term Supplementation with* all-rac*-*α*-Tocopheryl Acetate in a Randomized Controlled Trial

**DOI:** 10.1155/2016/6158436

**Published:** 2016-10-20

**Authors:** Alison M. Mondul, Steven C. Moore, Stephanie J. Weinstein, Anne M. Evans, Edward D. Karoly, Satu Männistö, Joshua N. Sampson, Demetrius Albanes

**Affiliations:** ^1^Department of Epidemiology, University of Michigan School of Public Health, Ann Arbor, MI, USA; ^2^Department of Health and Human Services, Division of Cancer Epidemiology and Genetics, National Cancer Institute, NIH, Nutritional Epidemiology Branch, Bethesda, MD, USA; ^3^Metabolon, Inc., Durham, NC, USA; ^4^Department of Chronic Disease Prevention, National Institute for Health and Welfare, Helsinki, Finland; ^5^Department of Health and Human Services, Division of Cancer Epidemiology and Genetics, National Cancer Institute, NIH, Biostatistics Branch, Bethesda, MD, USA

## Abstract

*Background*. The Alpha-Tocopherol, Beta-Carotene Cancer Prevention (ATBC) Study, a randomized controlled cancer prevention trial, showed a 32% reduction in prostate cancer incidence in response to vitamin E supplementation. Two other trials were not confirmatory, however.* Objective*. We compared the change in serum metabolome of the ATBC Study participants randomized to receive vitamin E to those who were not by randomly selecting 50 men from each of the intervention groups (50 mg/day all-*rac*-*α*-tocopheryl acetate (ATA), 20 mg/day *β*-carotene, both, placebo).* Methods*. Metabolomic profiling was conducted on baseline and follow-up fasting serum (Metabolon, Inc.).* Results*. After correction for multiple comparisons, five metabolites were statistically significantly altered (*β* is the change in metabolite level expressed as number of standard deviations on the log scale): *α*-CEHC sulfate (*β* = 1.51, *p* = 1.45 × 10^−38^), *α*-CEHC glucuronide (β = 1.41, *p* = 1.02 × 10^−31^), *α*-tocopherol (*β* = 0.97, *p* = 2.22 × 10^−13^), *γ*-tocopherol (*β* = −0.90, *p* = 1.76 × 10^−11^), and *β*-tocopherol (*β* = −0.73, *p* = 9.40 × 10^−8^). Glutarylcarnitine, beta-alanine, ornithine, and N6-acetyllysine were also decreased by ATA supplementation (*β* range 0.40 to −0.36), but not statistically significantly.* Conclusions*. Comparison of the observed metabolite alterations resulting from ATA supplementation to those in other vitamin E trials of different populations, dosages, or formulations may shed light on the apparently discordant vitamin E-prostate cancer risk findings.

## 1. Introduction

Vitamin E is a family of compounds that has long been thought to have biological properties that may prevent prostate and other cancers [1]. Of these compounds, *α*-tocopherol is the most bioavailable in humans and, according to the Institute of Medicine, is the only form of vitamin E that has been demonstrated to reverse vitamin E deficiency and is the most predominant form that is maintained in plasma and tissues [2]. Thus, *α*-tocopherol is the most thoroughly examined with respect to its potential beneficial health effects. The Alpha-Tocopherol, Beta-Carotene Cancer Prevention (ATBC) Study, a large randomized controlled trial of vitamin E and *β*-carotene supplementation in male Finnish smokers, found that vitamin E supplementation led to 32% lower prostate cancer incidence, particularly of more aggressive disease [3]. Results from two subsequent large controlled trials were not confirmatory, however, with the Selenium and Vitamin E Cancer Prevention Trial (SELECT) finding 17% higher prostate cancer incidence in men given vitamin E [4] and the Physicians' Health Study II (PHS II) showing no effect of vitamin E supplementation [5]. Several explanations for the divergent trial findings include the substantial difference in the *α*-tocopherol dose (ranging from 50 IU to 400 IU daily) and possible selective vitamin E-prostate cancer protective effects in cigarette smokers or for more advanced disease. Biologic mechanisms have also been investigated, including alterations in circulating androgens or vascular endothelial growth factors [6, 7], yet our understanding of the effects of *α*-tocopherol supplementation on risk of developing cancer remain incomplete.

Metabolomic profiling is a relatively new laboratory analytical tool that measures an array of low molecular weight biochemicals in a variety of matrices including blood [8]. Agnostic approaches such as genome-wide association studies have discovered new biological associations between specific genes or their biologic pathways and human disease, including cancer, as well as other phenotypes. Similarly, examination of the human metabolome offers the potential to discover molecular species relevant to various diseases [9–11], characteristics (e.g., energy balance and BMI) [12], and environmental exposures (e.g., diet, smoking, and vitamin supplementation) [13, 14]. Metabolomic profiling may also help elucidate how vitamin E supplementation impacted prostate cancer incidence in the aforementioned trials [15].

To our knowledge, only two studies have examined the biological response to supplementation with *α*-tocopherol in healthy individuals and both had very small sample sizes (*n* = 10) and short durations of supplementation (2 and 4 weeks, resp.) [16, 17]. One additional study examined patients with nonalcoholic steatohepatitis (NASH) who had been randomized to vitamin E or placebo and compared those whose disease responded to vitamin E to those who did not respond; this study also had a small sample size (*n* = 16 in each group) [18]. Our understanding of the biochemical effects of long-term *α*-tocopherol supplementation therefore remains incomplete. In the present study, we quantitatively examined the pre- and postenrollment fasting serum metabolome of 100 men randomized to receive long-term *α*-tocopherol supplementation and 100 men who did not receive *α*-tocopherol in the ATBC Study.

## 2. Methods

### 2.1. Study Population

The ATBC Study was a double-blind, placebo-controlled cancer prevention trial designed to test whether supplementation with *α*-tocopherol or *β*-carotene influenced cancer incidence [19]. Participants were recruited between 1985 and 1988 in southwestern Finland and were males between the ages of 50–69 years old and who smoked at least 5 cigarettes per day at enrollment. Trial participants (*n* = 29,133) were randomized to one of four groups based on a 2 × 2 factorial design: (1) *α*-tocopherol (*all-rac*-*α*-tocopheryl acetate (ATA), 50 mg/day), (2) *β*-carotene (20 mg/day), (3) both supplements, or (4) placebo. Supplementation continued for 5–8 years until the trial ended on April 30, 1993. Compliance which was estimated on the basis of residual capsule counts was excellent, with 80% of participants taking more than 95% of their capsules [19]. Overnight fasting blood samples were collected from all participants at baseline, and on-study samples were collected annually from a random sample of participants. The blood samples were processed to serum, aliquoted, and stored at −70°C. The ATBC Study was approved by institutional review boards at both the US National Cancer Institute and the Finnish National Public Health Institute; written informed consent was obtained from all trial participants.

For the present analysis, 50 men were randomly sampled from each trial intervention group for a total sample of 200 individuals; participants were classified as either receiving the trial *α*-tocopherol supplement (i.e., ATA alone or ATA plus *β*-carotene) or not (i.e., placebo or *β*-carotene alone). Men included in the current study sample had to (1) have both pre- and postrandomization fasting serum sample available with the follow-up sample collected at least 1 year after the baseline blood collection (mean = 3.3 years, range 1.0–6.9 years) and start of supplementation and (2) be cancer-free for at least 5 years after the follow-up blood collection. We required the participants to be cancer-free for 5 years after follow-up blood collection to minimize the possibility of reverse causation, that is, changes in the circulating metabolome resulting from undiagnosed cancer.

### 2.2. Metabolomic Analysis

Metabolomic profiling was conducted on baseline and follow-up serum samples at Metabolon, Inc. (Durham, NC), using an untargeted analysis capturing a broad array of low molecular weight compounds as described in detail previously [13, 20]. Briefly, samples were extracted and prepared for either UPLC/MS/MS^2^ or GC/MS. Raw data was extracted, peak-identified, and QC processed as described previously [20, 21]. For QA/QC purposes, additional samples were included with each day's analysis including extracts of a pool of well-characterized human serum, extracts of a pool created from a small aliquot of the experimental samples, and process blanks. QC samples were spaced evenly among the injections and all experimental samples were randomly distributed throughout the run with baseline and follow-up samples from each individual analyzed in the same batch. The median and interquartile range of the intraclass correlation coefficient (ICC) across the metabolites was 0.84 (0.52–0.96); these ICCs are similar to those seen previously in samples analyzed by this laboratory [22].

A total of 516 compounds were identified in our samples; any compound with more than three missing observations in either the baseline or follow-up sample was excluded, leaving 500 metabolites for analysis. Metabolites were then grouped into 9 mutually exclusive chemical classes (amino acids, carbohydrates, cofactors and vitamins, energy metabolites, lipids, nonstandard amino acids, nucleotides, peptides, and xenobiotics) based on the available literature [21].

### 2.3. Statistical Analysis

In order to account for batch variability, metabolite levels were divided by their batch's median value. Normalized metabolite levels were then log-transformed and missing values were imputed to the minimum of nonmissing values. In our primary analysis, we modeled the association between change in log-metabolite levels and trial assignment (i.e., ATA versus no ATA) by linear regression. Secondary analyses were conducted stratifying by number of cigarettes smoked per day, alcohol consumption, duration of supplementation with ATA, trial *β*-carotene supplementation, and baseline serum *α*-tocopherol concentration. The threshold for statistical significance for our main analysis was 0.0001022495 based on a Bonferroni correction for 500 tests to obtain a Family-Wise-Error-Rate (FWER) of 0.05 or 0.00021 based on a permutation correction. For the latter, we calculated the minimum *p* value for each of 10,000 permuted datasets, where we permuted the group assignment, and found the 0.05 quantile to be 0.00021. We further examined whether a particular chemical class of metabolites was over- or underrepresented among our top metabolites. For this analysis, we categorized metabolites as below a *p* value of 0.05 (yes/no) and as belonging to a chemical class (yes/no) and then used Fisher's exact test to determine whether the representation among the top metabolites was statistically significant at the threshold of 0.0056 based on a Bonferroni correction for 9 tests. All analyses were performed using SAS version 9.1.3 (SAS Institute, Cary, NC).

## 3. Results

Characteristics of the study population by trial ATA assignment are shown in Table 1. With the exception of 42% higher serum *α*-tocopherol concentrations at follow-up in vitamin E supplemented men, there were no differences by intervention arm, as would be expected based on the randomized design of the ATBC Study.

Table 2 presents findings for the 24 metabolites that changed in response to the ATA supplementation with a statistical significance of *p* < 0.05. After correction for multiple comparisons using a statistical threshold of *p* < 0.00021, five of the 500 metabolites identified in our analysis changed statistically significantly in response to vitamin E supplementation, that is, increases in *α*-CEHC sulfate, *α*-CEHC glucuronide, and *α*-tocopherol and reductions in *γ*-tocopherol and *β*-tocopherol (Table 2). The strength of these top vitamin E metabolite associations differed by neither smoking intensity (i.e., number of cigarettes smoked daily), with interaction *p* values for *α*-CEHC sulfate, *α*-CEHC glucuronide, *α*-tocopherol, *γ*-tocopherol, and *β*-tocopherol of 0.65, 0.11, 0.95, 0.63, and 0.87, respectively, nor subgroups of duration of ATA supplementation, alcohol consumption, baseline serum *α*-tocopherol concentration, or the trial *β*-carotene supplementation. One other vitamin E metabolite, gamma-CEHC-glucuronide, was more weakly related to ATA supplementation (beta-0.30).

Beyond the vitamin E-related compounds, the next most significant metabolites were four structurally similar amino acids—glutarylcarnitine, beta-alanine, ornithine, and N6-acetyllysine—that were decreased by ATA supplementation and shared fairly similar beta-estimates (−0.40 to −0.36) and fructose that increased in response. These associations were not, however, formally significant based on the *p* value threshold for multiple comparisons, and there were no statistically significant interactions between these or any other measured metabolites and ATA supplementation duration, smoking intensity, alcohol consumption, baseline serum *α*-tocopherol concentration, or the *β*-carotene supplement.

Examination of the chemical classes of the top metabolites revealed that the “cofactors and vitamins” class contained a greater number of significant metabolites (7 of 23, 30%, *p* = 3.45 × 10^−5^) than would be expected by chance alone. No other chemical class was over- or underrepresented in the most statistically significantly associated metabolites (all *p* > 0.10).

## 4. Discussion

In this randomized controlled trial of male smokers, we identified five metabolites whose relative concentrations changed significantly in response to long-term supplementation with* all-rac-α*-tocopheryl acetate (ATA). To our knowledge, this is the largest study and the only one of long-term supplementation to examine the change in the human serum or plasma metabolome in response to supplementation with this vitamin E compound. As anticipated and previously reported, *α*-tocopherol concentrations increased significantly in response to the ATBC trial vitamin E supplementation, while concentrations of *γ*-tocopherol and *β*-tocopherol decreased. Prior studies have shown such reductions in *γ*-tocopherol and *β*-tocopherol concentrations during *α*-tocopherol supplementation [23, 24]. *δ*-Tocopherol was not detected in our pre- or postsupplementation samples in either intervention group.

The two strongest effects on serum metabolites we observed were substantially elevated concentrations of *α*-CEHC sulfate and *α*-CEHC glucuronide. *α*-CEHC is an end-product *α*-tocopherol metabolite generated through serial *ω*-oxidation and *β*-oxidation by cytochrome P450 (CYP) enzymes, particularly CYP4F2, CYP3A4, and CYP3A5, which may be upregulated by all vitamin E compounds through activation of the nuclear pregnane X receptor (PXR), although the role of PXR remains controversial [25, 26]. These metabolic products are subsequently glucuronidated or sulfated for excretion by UDP-glucuronosyltransferases (UGT) and sulfatases, respectively [27], which appear to occur once an individual's capacity for hepatic incorporation of *α*-tocopherol into low-density lipoprotein (LDL) particles has been reached. Based on this, circulating *α*-CEHC has been suggested as a biomarker of optimum *α*-tocopherol status [28, 29]. This suggests that the circulating and tissue-specific concentrations of *α*-CEHC and its glucuronide and sulfate metabolites are dependent upon the dose of *α*-tocopherol administered, as well as an individual's LDL concentrations and level of oxidative stress [28]. In fact, cigarette smoking, which increases the latter, has been associated with *α*-CEHC concentrations [30]. Interestingly, *α*-CEHC may have independent anticarcinogenic effects that *α*-tocopherol does not exhibit, including being strongly anti-inflammatory [31]. This may be consistent with the hypothesis that the conflicting prostate cancer clinical trial findings for *α*-tocopherol supplementation could be due to either the different vitamin E doses administered or the cigarette smoking status of the trial populations. An inverse association was observed between vitamin E supplementation and risk of prostate cancer in the ATBC Study, which administered 50 IU/day and included only men who were current smokers at enrollment [3]. In contrast, there was no supplement effect in the PHS II which administered 400 IU every other day and included mostly nonsmokers [5], and higher prostate cancer incidence was observed in SELECT among mostly nonsmoking men receiving 400 IU daily [4]. To our knowledge, neither circulating *α*-CEHC nor its conjugated metabolites have been examined in relation to risk of prostate or other cancers.

Two previous studies of metabolomic profile changes in response to *α*-tocopherol supplementation in healthy participants did not identify any of the top five serum metabolites observed here [16, 17], possibly due to one or more differences in the study methodologies. For example, both prior studies supplemented participants for only 14 days, as compared with an average of 3.3 years in the present analysis. Also, the *α*-tocopherol doses and preparations differed greatly from the ATBC Study, with one administering 400 mg of* RRR-α*-tocopheryl acetate daily [17] and the other feeding participants 55 g of almonds for 7 days followed by 600 IU/day of* all-rac*-*α*-tocopheryl acetate (with no washout period) [16]. Our sample size was much larger than either of the previous studies; that is, *n* = 200 individuals (100 each in the ATA and no ATA groups) as compared with 10 vitamin E-supplemented individuals in each prior study (and no placebo control groups). The metabolomic assay platforms also differed across the three studies. The study comparing metabolomic profiles in NASH patients who did and did not respond to vitamin E supplementation also did not identify any of our top metabolites as being associated with vitamin E treatment response [18].

Although not formally statistically significant, three structurally similar amino acids—beta-alanine, ornithine, and N6-acetyllysine—as well as glutarylcarnitine, decreased during ATA supplementation. Whether serum changes in these metabolites are related to the oxidative degradation of the ATA phytyl side-chain (e.g., through upregulation of CYP3A activity) or to an effect of the resulting two-carbon acetyl groups on fatty acid and possibly acetyl-coenzyme A, metabolism is not known. Exploration of the pathway(s) through which vitamin E impacted these biochemical alterations could inform our understanding of both vitamin E metabolism and the effect of vitamin E on prostate carcinogenesis.

Strengths of the present analysis include the parent trial's randomized controlled design and our measurement of metabolomics profiles both before and after supplementation in both the supplemented and unsupplemented groups. We employed a relatively large sample size, participants were supplemented for at least a year and an average of more than three years, and serum was obtained after an overnight fast. Study limitations included an all Caucasian male smoker population and the ability to test only one dose and preparation of *α*-tocopherol based on the parent ATBC trial.

## 5. Conclusions

In this analysis of presupplementation versus on-study serum metabolomic profiles nested within a large controlled trial of vitamin E, we found the presence of several molecules that were quantitatively altered by chronic supplementation with ATA, including two conjugated metabolites of *α*-CEHC. Further study of *α*-CEHC and its metabolites in relation to risk of prostate and other cancers in humans is warranted; in particular, examination of which enzymes (e.g., CYPs, UGTs, or sulfatases) may be upregulated during supplementation with *α*-tocopherol. In addition, comparison of pre- and postsupplementation metabolomic profiles between this study and other clinical trials of *α*-tocopherol supplementation conducted in different populations or using different dosages or preparations (e.g., SELECT) may shed light on the apparently discordant prostate cancer findings across the trials.

## Figures and Tables

**Table 1:** Participant characteristics^1^ by ATA assignment in the ATBC Study.

	No ATA	Yes ATA	*p* value
*N*	100	100	
Age (years)	57.5 (4.9)	57.8 (5.0)	*0.70*
Height (cm)	174 (5.9)	174 (6.6)	*0.91*
Weight (kg)	79.6 (10.7)	78.4 (12.5)	*0.40*
BMI (kg/m^2^)	26.2 (3.2)	25.8 (3.5)	*0.36*
Cigarettes per day	20.3 (9.9)	19.8 (8.9)	*0.80*
Years of smoking	35.1 (9.6)	35.7 (9.5)	*0.68*
Quit smoking at follow-up visit (%)	29.0	22.0	*0.21*
Physically active (%)	23.0	25.0	*0.74*
>Elementary school education (%)	29.0	23.0	*0.33*
Dietary intake per day			
Total energy (kcal)	2,775 (691)	2,763 (695)	*0.60*
Fruit (g)	235 (204)	226 (161)	*0.80*
Vegetables (g)	305 (113)	319 (130)	*0.80*
Red meat (g)	75.0 (31.4)	75.6 (29.3)	*0.71*
Alcohol (ethanol, g)	13.5 (16.8)	14.7 (16.0)	*0.19*
Serum concentrations			
Total cholesterol (mmol/L)			
Baseline	6.4 (1.0)	6.4 (1.1)	*0.87*
Follow-up	6.0 (1.0)	6.3 (1.2)	*0.20*
ATA (mg/L)			
Baseline	12.5 (3.1)	12.1 (2.9)	*0.33*
Follow-up	12.9 (3.2)	18.3 (4.2)	*<0.0001*
*β*-Carotene (*μ*g/L)			
Baseline	272 (255)	240 (123)	*0.76*
Follow-up	1,887 (1,886)	2,014 (2,073)	*0.72*
Retinol (*μ*g/L)			
Baseline	576 (107)	585 (122)	*0.82*
Follow-up	597 (120)	614 (136)	*0.41*

^1^All variables are from the baseline questionnaire unless otherwise indicated. Values are means (standard deviation) unless otherwise indicated.

**Table 2:** Metabolites that changed significantly (*p* < 0.05) in response to ATA supplementation in the ATBC Study, ranked by statistical significance (smallest to largest *p* value).

Metabolite	Chemical class	Number > LOD^*∗*^ at baseline	Number > LOD^*∗*^ at follow-up	Effect size (*β*)^†^	*p* value
Alpha-CEHC sulfate	Cofactors and vitamins	39	151	1.512	1.45 × 10^−38^
Alpha-CEHC glucuronide	Cofactors and vitamins	4	87	1.412	1.02 × 10^−31^
Alpha-tocopherol	Cofactors and vitamins	200	200	0.974	2.22 × 10^−13^
Gamma-tocopherol	Cofactors and vitamins	196	188	−0.902	1.76 × 10^−11^
Beta-tocopherol	Cofactors and vitamins	199	186	−0.731	9.40 × 10^−8^
Fructose	Carbohydrate	200	200	0.401	0.0043
Glutarylcarnitine (C5)	Amino acid	200	200	−0.400	0.0044
Beta-alanine	Amino acid	200	200	−0.386	0.0061
Ornithine	Amino acid	200	200	−0.363	0.0100
N6-acetyllysine	Amino acid	200	200	−0.357	0.0112
Gamma-glutamylisoleucine	Peptide	200	200	−0.332	0.0185
2-Ethylhexanoate	Xenobiotics	200	200	0.316	0.0253
Bilirubin (E,Z or Z,E)^*∗*^	Cofactors and vitamins	184	190	0.312	0.0268
Seryl-leucine	Peptide	179	184	−0.306	0.0300
Gamma-CEHC glucuronide	Cofactors and vitamins	90	127	0.303	0.0319
Bradykinin, des-arg(9)	Peptide	192	194	0.303	0.0320
Arabinose	Carbohydrate	117	165	−0.302	0.0325
3-Hydroxydecanoate	Lipid	200	200	0.301	0.0327
Linolenate [alpha or gamma; (18:3n3 or 6)]	Lipid	200	200	0.301	0.0328
Phenylalanylarginine	Peptide	133	158	0.297	0.0352
3-Hydroxypropanoate	Lipid	200	200	−0.297	0.0355
Inositol 1-phosphate (I1P)	Lipid	200	200	0.293	0.0377
5 alpha-androstan-3 alpha,17 beta-diol disulfate	Lipid	183	171	0.287	0.0425
Creatinine	Amino acid	200	200	−0.277	0.0496

^*∗*^LOD = limit of detection.

^†^Effect size denotes the change in metabolite level (expressed in number of standard deviations on the log scale) for the ATA arm versus the no ATA arm. The effect size and *p* value were estimated by linear regression.

## Abbreviations

ATBC Study: Alpha-Tocopherol, Beta-Carotene Cancer Prevention Study
CYP: Cytochrome P450
FWER: Family-Wise-Error-Rate
GC: Gas chromatography
ICC: Intraclass correlation coefficient
LOD: Limit of detection
MS: Mass spectroscopy
MS^2^: Tandem mass spectroscopy
PHS II: Physicians' Health Study II
QC: Quality control
SELECT: Selenium and Vitamin E Cancer Prevention Trial
UGT: UDP-glucuronosyltransferase
UPLC: Ultrahigh performance liquid chromatography
ATA: all-rac-*α*-Tocopheryl acetate.

## Data Access

Data will not be shared to preserve the confidentiality of the ATBC Study participants.
